# Thermopneumatic Soft Micro Bellows Actuator for Standalone Operation

**DOI:** 10.3390/mi12010046

**Published:** 2021-01-01

**Authors:** Seongbeom Ahn, Woojun Jung, Kyungho Ko, Yeongchan Lee, Chanju Lee, Yongha Hwang

**Affiliations:** Department of Electro-Mechanical Systems Engineering, Korea University, Sejong 30019, Korea; ahnsb98@korea.ac.kr (S.A.); wjkst2010@korea.ac.kr (W.J.); gokyungho123@korea.ac.kr (K.K.); liws214@korea.ac.kr (Y.L.); cjl1226@korea.ac.kr (C.L.)

**Keywords:** thermopneumatic, micro actuator, soft bellows actuator, independent actuation, polydimethylsiloxane, 3D printing, standalone

## Abstract

Typical pneumatic soft micro actuators can be manufactured without using heavy driving components such as pumps and power supplies by adopting an independent battery-powered mechanism. In this study, a thermopneumatically operated soft micro bellows actuator was manufactured, and the standalone operation of the actuator was experimentally validated. Thermopneumatic actuation is based on heating a sealed cavity inside the elastomer of the actuator to raise the pressure, leading to deflection of the elastomer. The bellows actuator was fabricated by casting polydimethylsiloxane (PDMS) using the 3D-printed soluble mold technique to prevent leakage, which is inherent in conventional soft lithography due to the bonding of individual layers. The heater, manufactured separately using winding copper wire, was inserted into the cavity of the bellows actuator, which together formed the thermopneumatic actuator. The 3D coil heater and bellows allowed immediate heat transfer and free movement in the intended direction, which is unachievable for conventional microfabrication. The fabricated actuator produced a stroke of 2184 μm, equivalent to 62% of the body, and exerted a force of 90.2 mN at a voltage of 0.55 V. A system in which the thermopneumatic actuator was driven by alkaline batteries and a control circuit also demonstrated a repetitive standalone operation.

## 1. Introduction

Soft actuators are manufactured from soft materials with high compliance and degrees of freedom to handle unpredictable and dynamic tasks in unstructured environments [1,2,3]. The introduction of soft materials such as polydimethylsiloxane (PDMS) [4], polyurethane (PU) [5], and thermoplastic polyurethane (TPU) [6], which have the advantages of elasticity, easy processing (e.g., injection or casting), and low cost, inherently provides greater flexibility in comparison to the typical robot, which is made of a rigid material [7]. Fang et al. produced an assistive device that helped knee rehabilitation training using TPU fabric. Calderón et al. developed a soft robot composed of a silicone elastomer (Ecoflex 00-50) by mimicking an earthworm [8,9]. Wang et al. reported a soft grasping robot with a hinge using PDMS. In particular, PDMS has been used in biomedical applications that include lab-on-a-chip that deals with biological specifications and wearable devices attached to the skin, thereby proving its biocompatibility over the past decades [10,11,12,13].

Various driving methods have been developed for soft actuators, which include pneumatic pressure, cable-driven tendon, and electroactive polymer (EAP) [14]. Pneumatic actuation applies air pressure to inflate a membrane with a low Young’s modulus; therefore, it requires an external drive source such as a compressor, pump, or syringe [15,16]. The cable-driven actuation produces a bend in the actuator when pulling the bendable cable embedded in the soft actuator by an external motor [17]. EAP is a soft material that generates mechanical deformation when stimulated by an electric field. However, to produce a noticeable deformation, a high voltage of several kV is required, for which a bulky power supply is required [18]. Thermopneumatic soft actuators are being developed that increase pressure by heating the internal air or fluid with built-in heaters, thereby switching to mechanical strokes [19]. Here, the thermopneumatic actuators can be configured by heaters and power supplies so that they offer favorable alternatives for miniaturization and standalone operation to relieve the payload without the need for additional devices for driving.

In this study, the micro thermopneumatic bellows actuators with standalone drives were designed, constructed, and evaluated by miniaturizing the 3D bellows actuators developed primarily for pneumatic driving and inserting 3D heaters for low voltage operation. The heater supplies heat energy by using Joule heating when the current passes through a conductive wire. The thermal energy supplied to the sealed internal air of the bellows actuator increases pressure and exerts force from the inside of the bellows actuator, generating movement in the intended direction as the folded bellows expands.

To manufacture the micro bellows actuator, the 3D-printed soluble mold technique was used to realize the fully-3D micro body beyond the limited 3D (2.5D) [20] structure of the conventional soft lithography technique [21,22]. Because the process of stacking separate thin structures is excluded, this fabrication technique, which uses sacrificial molds printed with build and support materials to cast liquid polymer, has the ability to minimize unnecessary bonding while also achieving the desired 3D structure.

It is desirable that the microheater takes a 3D shape so that it can extend the heating area to effectively heat the inner space of the micro bellows actuators. Therefore, the thermopneumatic soft micro bellows actuator was implemented by inserting a heater manufactured by winding a copper wire in the form of a 3D coil having a low resistivity for low-voltage driving. To analyze the thermodynamic and mechanical responses of the actuator, the temperature of the internal cavity was estimated by measuring the resistance change of the heater. The fabricated thermopneumatic bellows actuator was operated by an electric current to the heater using a direct current (DC) power supply, and the displacement and force were measured according to the rise in temperature of the sealed cavity. Finally, the standalone operation of the proposed thermopneumatic bellows actuator was demonstrated using an embedded control circuit driven by batteries.

## 2. Design and Fabrication

### 2.1. Design

**Figure 1:**
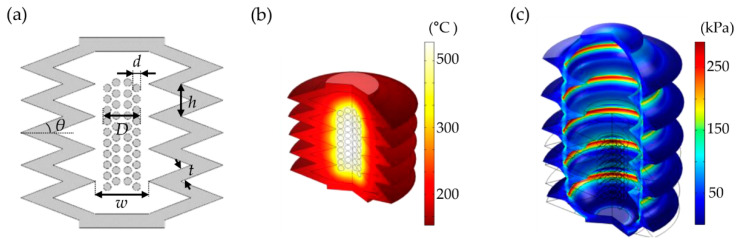
Concept of thermopneumatic bellows actuator. (**a**) Schematic cross-section showing the main design parameters of bellows angle (*θ* = 20°), bellows thickness (*t* = 250 μm), bellows height (*h* = 655 μm), wire diameter (*d* = 200 μm), coil heater diameter (*D* = 1000 μm), and bellows inner diameter (*w* = 1100 μm). The coil is double wound inside the bellows. (**b**) Temperature distribution of thermopneumatic bellows actuator using conjugate heat transfer module. The results are obtained by setting the power of the heater to 0.98 W, ambient temperature to 20 °C, and convection heat transfer coefficient to 5 W/(m^2^∙K). (**c**) Simulated deflection plots of the thermopneumatic bellows actuator using solid mechanics module. Wire frame outlines the resting actuator. Deflected actuator is shown with a color map depicting von Mises stress. (Red and blue indicate 280 kPa and 0 Pa, respectively.) The second-order Ogden model was used to evaluate the mechanical motion of PDMS bellows.

To fully describe the design and operational principle of the proposed device, Figure 1 shows the structure of the thermopneumatic bellows actuator used in this work with the design parameters. Among the design parameters of the cross-section, as shown in Figure 1a, the bellows angle (*θ*) was determined to be 20° with the largest displacement ratio estimated by a numerical simulation performed by varying the bellows angle between 10° and 30°, as shown in the previous study [23]. The bellows thickness (*t*) for thermopneumatic actuator was 250 μm, considering the empirical limitations of the 3D printing and casting process. Polyamide-imide enameled copper wire of a diameter (*d*) of 200 μm was used for coil heaters. The coil heater diameter (*D*) was set to 1000 μm by the wire diameter and winding method of the copper wire, which is discussed in Section 2.2. Finally, the bellows inner diameter (*w*) was designed to be 1100 μm to prevent interference caused by contact with the heater during operation. The bellows actuator had five repeated bellows.

The working principle of the thermopneumatic actuation is that the internal air of the bellows actuator expands when heated by a heater, causing deflection of the bellows. The thermodynamic relation between the absolute temperature *T*, pressure *P* and volume *V* of a system is given by the well-known ideal gas equation:

(1) PV=nRT

where *n* is the number of moles of the gas and *R* is the gas constant. The variation of volume and pressure by changing the temperature is then given by:

(2) $$\frac{P_{0}V_{0}}{T_{0}}=\frac{P_{1}V_{1}}{T_{1}}$$

where *V*_1_ and *P*_1_ are the volume and pressure at a set temperature *T*_1_, whereas *V*_0_ and *P*_0_ are the volume and pressure at room temperature *T*_0_, which is 293.15 K. As the temperature increases, the volume of the air expands and the pressure of the air increases causing a deformation in the actuator. Therefore, the force *F* exerted by the bellows actuator is expressed as:

(3) $$F=(P_{1}-P_{0})S=\left(\frac{P_{0}V_{0}T_{1}}{V_{1}T_{0}}-P_{0}\right)S$$

where *S* is the effective area where the bellows head contacts the object. Since PDMS is hyperelastic, the pressure of the cavity according to the ideal gas law does not accurately account for the changes in PDMS features when it expands [24].

For numerical analysis of the motion of the designed thermopneumatic bellows actuator, COMSOL Multiphysics^®^ (Stockholm, Sweden) was used to calculate the heat distribution of the entire actuator by the heater using the conjugate heat transfer module. The mechanical motion according to the thermal expansion of the air was sequentially calculated using the solid mechanical module given in Figure 1b–c. Using a simplified heater with an axisymmetric structure, the movement of the actuator was recorded as the maximum displacement when it reached the steady state which was 60 s after supplying power to the Joule heater through a time-dependent study. In the heat distribution analysis, the heat transfer coefficient of the convective heat flux generated on the bellows surface was set to 5 W/(m^2^∙K), and the ambient temperature was set to room temperature (20 °C).

When heated at high speed, PDMS was reported to be thermally decomposed at 530 °C [25]. When the heater applied 0.98 W of power to produce the highest heat within the limit of the PDMS deformation, the maximum temperature of the heater and the inner PDMS surface of the bellows were estimated to be 541.87 °C and 421.77 °C, respectively. In addition, the average temperature of the heated air of the entire cavity was 324.99 °C, resulting in a pressure of 12.62 kPa, which was consistent with previously reported work [23]. As shown in Figure 1c, the pressure generated by thermal expansion caused the soft PDMS bellows to unfold. In other words, the von Mises stress of 281.50 kPa was applied to the edge of the folded bellows, operating vertically against the direction in which the bellows were folded, and consequently resulting in the intended stroke of 1976 μm.

### 2.2. Fabrication

**Figure 2:**
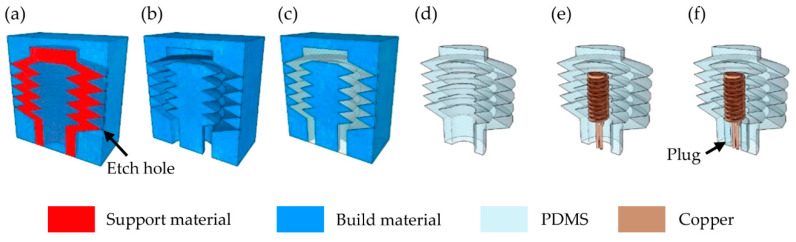
Fabrication process of cross-sectional thermopneumatic bellows actuator. (**a**) The actuator was printed through a 3D printer as a mold composed of build and support material. (**b**) Support material was dissolved through etch holes placed around the edge. (**c**) Polydimethylsiloxane prepolymer was poured and cured after vacuum. (**d**) PDMS bellows actuator after dissolving the mold. (**e**) 3D coil heater implanted inside the bellows actuator. (**f**) A thermopneumatic actuator after sealing with the PDMS plug.

The thermopneumatic bellows actuator was fabricated using a technique of chemically removing the mold after casting PDMS, an elastomer [26]. As shown in Figure 2a, after designing with 3D CAD (Inventor^®^, Autodesk, Mill Valley, CA, USA) the mold was printed with a 3D printer (3Z STUDIO, Solidscape, Merrimack, NH, USA) using a build material and a support material that filled the empty space of the mold to support the structure during stacking of each printed layer. The printed output was dipped in a dewaxing solvent (BIOACT^®^ VSO, Petroferm, Warren, NJ, USA) that flowed at 400 rpm at 50 °C and selectively removed the support material for 18 h, after which it was dried for 3 h in a vacuum chamber as shown in Figure 2b. There were 20 etch holes connecting the outer walls of the hexahedron mold with the edges of the inner bellows. The etch hole was designed to be 400 μm × 200 μm to help with the inflow of the dewaxing solvent but to not interfere with the appearance of the bellows. In Figure 2b, the central part was connected and fixed to the outer shell structure by supporting bridges (not represented in the figure) located at the bottom of the mold.

A PDMS prepolymer (Sylgard 184, Dow Corning, Midland, MI, USA), which was mixed with a curing agent in a 15:1 weight ratio, was placed into a vacuum chamber to remove air bubbles, and then poured into the empty space of the mold. The mold filled with PDMS was degassed again inside the vacuum chamber for 2 h in order that PDMS could be fully filled inside the mold, and then it was cured in an oven at 75 °C for 24 h (Figure 2c). Next, the mold was placed in acetone for 3 h to dissolve, followed by cleaning with deionized (DI) water, after which a single body bellows actuator consisting of PDMS was formed (Figure 2d). It should be noted that the 3D-printed soluble mold technique fabricated 3D elements in a single body by excluding aligning and bonding steps of soft lithography, which were required for multiple separate layers. In addition, the use of 3D printers allowed faster prototyping than the typical microfabrication based on the soft lithography.

The Joule heater located inside the actuator was made of copper wire covered with enamel. Although several types of commercially available copper wires were tested, copper wires with a diameter of 0.15 mm or less, heated up to approximately 300 °C and broke. The Joule heater made of a 0.2 mm diameter copper wire maintained the mechanical stiffness for uniform heat transfer at the center of the bellows and retained a 3D coil shape without bending. It was also experimentally confirmed that the heater was reusable after the electric power was applied at the required temperature for the actuator. After the copper wire with a diameter of 0.2 mm was cut to a length of 50 mm to maintain a resistance of 50 ± 1 mΩ, the coil heater was formed by double winding the copper wire around a 0.2 mm diameter stainless steel pole, with an outer coil diameter of 1.0 mm. The manufactured heater was used as the actuator element only if the resistance measured using a milliohmmeter (MO-2012, LUTRON, Taipei, Taiwan) was uniformly within ±2%.

The 3D heater supplying the heat energy to the cavity inside the bellows actuator was inserted 5.0 mm from the bellows entrance and placed in the center of the cavity (Figure 2e). To tightly block the bellows entrance after the heater was inserted, a cylinder-shaped PDMS plug with an outer diameter equal to the inner diameter of the actuator entrance was manufactured separately and inserted into the bellows entrance. The plug had an oval center hole with a 0.4 mm long axis that allowed the heater to be connected externally. The liquid PDMS was covered with the gap between the actuator and the plug and cured in an oven at 75 °C for 30 min to seal the cavity of the actuator (Figure 2f).

**Figure 3:**
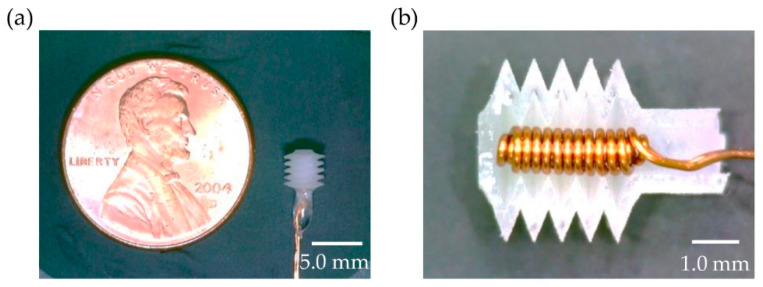
(**a**) Fabricated thermopneumatic bellows actuator. (**b**) Cross-section of the bellows actuator with the coil heater.

The fabricated thermopneumatic bellows actuator using the 3D-printed soluble mold technique is shown in Figure 3. The micro thermopneumatic bellows actuator has a volume of 26.4 mm^3^ and mass of 42 mg, which could operate as a thermopneumatic mechanism by applying low power at the level supplied by a battery. By cutting the fabricated actuator, as shown in Figure 3b into a plane perpendicular to the entrance, it was confirmed that the heater did not physically interfere with the bellows edges.

## 3. Experimental Results

### 3.1. Temperature Characteristics

**Figure 4:**
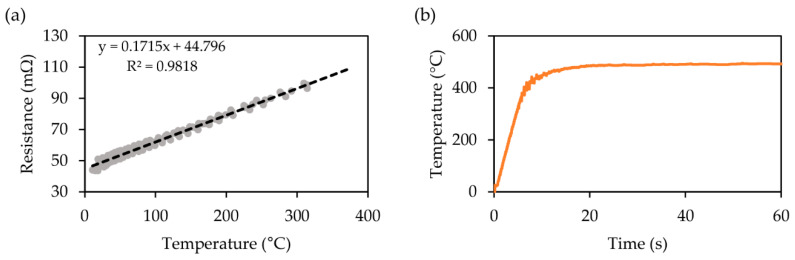
Temperature characteristics of a microheater. (**a**) Calibration of microheater through linear regression. R-squared of the linear regression is 0.9818. (**b**) Temperature–time curves under voltages of 0.5 V, measured based on the relationship between the resistance and temperature of the heater.

Methods for temperature detection are divided into contact and non-contact types [27]. The non-contact temperature sensors, such as infrared (IR) thermometers, can only measure the surface temperature of an object. Because the cavity inside the thermopneumatic bellows actuator is surrounded by PDMS, the IR thermometer has a limitation in accurately measuring the temperature of the closed air in the cavity. On the other hand, contact temperature sensors such as thermocouples, resistance temperature detectors, and thermistors that require insertion inside the actuator cause the size of the actuator to increase owing to the space occupied by the sensor, and further require additional equipment to read the sensor. Alternatively, the temperature of the cavity with a volume of 12.7 mm^3^ inside the micro actuator was analyzed by measuring the temperature of the heater itself because the cavity was heated to the same level as the heater in 1.52 s. To measure the temperature of the heater, it was experimentally matched with the relationship of the resistance and temperature of the heater before inserting the heater into the actuator. R-T (resistance-temperature) relationship of the heater was measured by employing a linear regression on the collected data from an IR camera (Seek Thermal Compact PRO, Santa Barbara, CA, USA) for the temperature and a digital multimeter (TX3 True RMS Digital Multimeter, Tektronix, Beaverton, OR, USA) for the resistance outside the bellows actuator (Figure 4a). The resistance of the heater was 48 mΩ at room temperature (20 °C) and 96 mΩ at 300 °C. Consequently, the heater temperature above 300 °C, which was outside the measurement range of the IR camera, was estimated using the experimental relationship between the resistance and the temperature of the heater given R = 0.1715 T + 44.796. After inserting the heater into the bellows actuator, it was possible to measure the internal temperature of the bellows in real time through the resistance of the heater, as shown in Figure 4b. When 0.50 V was applied to the heater, the heater inside the bellows actuator was heated to 492 °C, and 12.6 s was taken to reach a steady state. The resistance of the heater according to the temperature was used to analyze the mechanical motion of the actuator using COMSOL^®^ and to reduce the discrepancy with the measurement result.

### 3.2. Displacement Characteristics

**Figure 5:**
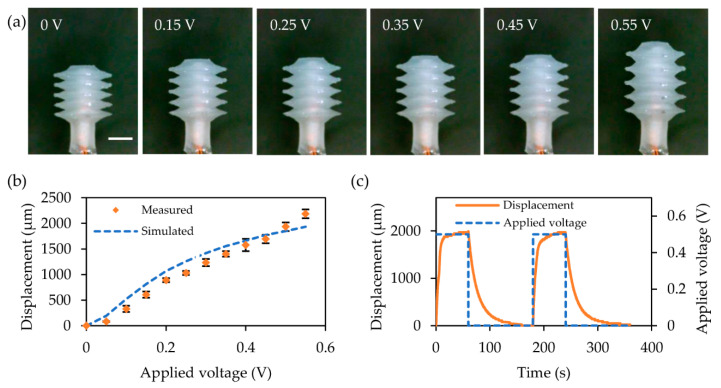
Displacement response generated by Joule heating. (**a**) Images of a thermopneumatic bellows actuator in stop motion due to a voltage applied at 0.10 V intervals from 0 V to 0.55 V. The scale bar is 2.0 mm. (**b**) Steady-state displacement of thermopneumatic bellows actuator versus applied voltage to the heater. Voltage was applied to the heater at intervals of 0.05 V up to the maximum operating voltage of 0.55 V. (**c**) Displacement of thermopneumatic bellows actuator when two cycles of 0.50 V pulse voltage with 180 s, duty ratio of 33% were applied.

Figure 5 shows the increased displacement of the thermopneumatic bellows actuator by applying a voltage of 0.55 V at 0.05 V intervals. It was observed when a voltage of 0.60 V or more was applied to the heater, a pressure exceeding the ultimate tensile strength of PDMS was generated inside the actuator, resulting in damage to the bellows actuator. The displacement, therefore, was measured for a voltage less than 0.60 V. The displacement was measured perpendicular to the plane from the head of the bellows using an optical microscope (UM12, Microlinks Technology, Kaohsiung, Taiwan), and then the recorded image frames were analyzed using a custom-built Python-based image processing tool. The expected and measured displacements as a function of the supplied voltage are plotted in Figure 5b. When a voltage of 0.55 V was applied, the actuator showed a displacement of average of 2184 μm from the five samples. There was an average error of 16.2% compared to the predicted value. This is most likely because the measured performance of the actuator is affected by the mixing ratio of the base and agent mixture of PDMS. Also, the elastic properties of the fabricated PDMS have a discrepancy owing to the inconsistency with the parameters of the hyperelastic Ogden model for the numerical analysis [28,29].

The movement of the actuator reached a steady state after 15.24 s of applying a constant voltage. Therefore, in order to verify the repeated operation of the thermopneumatic bellows actuator, the displacement was measured by applying a pulse of 0.50 V with a 33% duty ratio with a heating time of 60 s (including a retention driving) and a cooling time of 120 s (Figure 5c). The displacement took 2.69 s longer to reach the steady-state response compared to the temperature of the inner heater using the heater resistance, as shown in Figure 4b. This is consistent with the calculated result that it took an average of 1.52 s for the air in the cavity to heat up to the same temperature as the heater. Therefore, it was confirmed that the increased temperature of the heater immediately heats the air in the actuator cavity, resulting in the mechanical motion of the actuator. It was observed that the actuator showed a 9.4% decrease in displacement after 10 repetitive operations, and a 12.8% decrease after 15 repetitions. The thermopneumatic driving method inherently increases the temperature of the PDMS bellows, causing a decrease in the ultimate tensile strength [30]. Thus, it is most likely that degradation by heat caused a decrease in performance of the actuator.

In this paper, we built the bellows actuators based on PDMS, which has been verified for decades for biomedical applications. However, considering only thermal degradation, materials that are more thermally stable than PDMS such as α, ω-Trimethylsiloxy-poly(dimethyl-methylchloromethyl) siloxane (PDCMS) and polydimethylsiloxane/zeolitic imidazolate framework (PDMS/ZIF) can be applied for the thermopneumatic bellows actuators [31,32]. Changes in the design parameters of the actuator can also result in thermally stable behavior of a specific stroke. As the number of bellows increases, the displacement of the bellows actuator increases. Thus, by driving at a voltage lower than the voltage that could cause the thermal degradation and also increasing the number of bellows sufficient to achieve the required stroke, the exposure to high temperatures in PDMS can be reduced, thereby preventing the reduction of the ultimate tensile strength in PDMS. More importantly, the 3D-printed soluble mold technique, which is developed to produce a bellows actuator, readily enables a variety of soft polymer actuators or design changes without fabrication process revision, except for selecting the appropriate dissolving solvent [23].

Several studies have reported the PDMS membrane with thickness ranging from tens of μm to hundreds of μm to pneumatic actuation without side effects from gas permeation [10,33]. In particular, according to S Sawano, the 110 μm thick PDMS membrane pump exhibits only about 2% deflection change over 1 h and about 13% over 10 h due to the gas leakage [33]. Since the bellows actuator in this paper consists of a PDMS membrane (250 μm in thickness) that is more than twice as thick, it is relatively insensitive to the gas leakage caused by the pores inside the PDMS. In addition, the bellows actuator maintains the internal cavity pressure continuously by the supply of the electrical power for the required displacement. After the driving power is switched off, the pressure inside the cavity is lowered by air temperature cooling, not by the leakage, which in turn the actuator is restored to its original state. As a result, the operation of the bellows actuator is governed by the pressure formed by the electrical power supplied, while the gas leakage is negligible.

### 3.3. Force Characteristics

**Figure 6:**
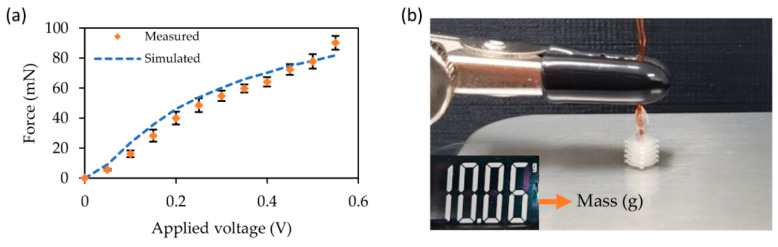
Steady-state force characteristics. (**a**) Experimental data (dot) and model prediction (solid line) of the thermopneumatic actuator, voltage was applied up to the maximum operating voltage of 0.55 V, and the force was measured at 0.05 V intervals. (**b**) Measuring the vertical force applied during expansion when 0.55 V, the maximum operating voltage of the thermopneumatic actuator, is applied. Inset shows measured mass in gram.

As shown in Figure 6, the force exerted by the actuator in the stroke direction according to the driving voltage was compared with the expected result. The tail of the actuator was fixed to the clamp, and the head, which was the part where the actuator deformed, was positioned such that it touched an electronic scale. The measured weight according to the force applied vertically by the actuator’s expanding head was calculated as the exerted force by multiplying with the acceleration due to gravity. As the internally applied pneumatic pressure was applied to the walls of the inner space of the actuator, it was directly transmitted in the form of force exerted by the bellows actuator, resulting in a linear response. The measured force had an average error of 12.2% when compared with the numerically calculated force. It seemed that the heat conduction occurred between the end of the bellows and the thermal conductive surface of the scale, thus resulting in a discrepancy between them. The exerted force of 90.2 mN demonstrated that a single actuator with a thermopneumatic actuation mechanism has sufficient force to push an object [34].

### 3.4. Standalone Operation of Thermopneumatic Bellows Actuators

**Figure 7:**
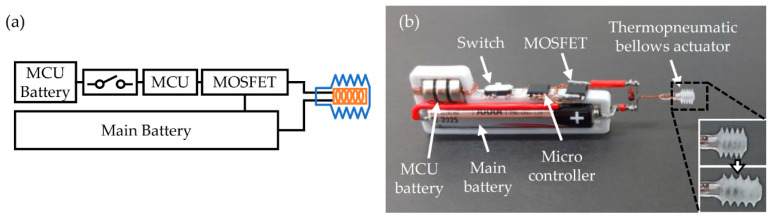
Independent drive system of thermopneumatic actuator. (**a**) Schematic diagram of the autonomous system for standalone operation of the thermopneumatic bellows actuator. (**b**) Photograph of an independent circuit (bird’s eye view): button cell battery for MCU, MOSFET for switching, MCU for controlling the signal, a 1.5 V alkaline battery for supplying power to the heater.

The power supply can provide stable voltage and current to drive the thermopneumatic bellows actuator. However, compared to the entire volume and weight of the micro actuator, the commercially available power supply is generally heavy. In addition, the external power units must be wired to an outlet. To relieve the bulky payload, a thermopneumatic bellows actuator system for standalone driving was built with batteries and a control circuit. The standalone system depicted in Figure 7 and Video S1 is composed of an MCU (ATTINY13A-SSU, Microchip Technology, Chandler, AZ, USA), a switching MOSFET (FDS6680A, Fairchild Semiconductor, Sunnyvale, CA, USA), button cell batteries (LR732), and an alkaline battery. To supply a voltage of 0.40 V to the heater, a 1.5 V AAAA battery for the heater drive was connected to the thermopneumatic bellows actuator through the switching MOSFET.

## 4. Conclusions

Soft micro bellows actuators with thermopneumatic actuation for independent driving were designed, fabricated, and evaluated. The 3D thermopneumatic bellows actuator was manufactured using the 3D-printed soluble mold technique in a single body; therefore, no degradation of performance due to air leakage occurred until the fracture stress of the PDMS itself was reached. The performance of the actuator was evaluated by supplying a voltage of up to 0.55 V to prevent the thermal degradation of PDMS. It was observed that the thermopneumatic bellows actuator showed displacement up to 2184 μm and operated repeatedly. It was also confirmed that a standalone operation was possible by configuring the actuator with a control circuit and batteries instead of bulky external devices by changing the operation mechanism from pneumatic to thermopneumatic method. This implies that an autonomous micro soft robot can be constructed that achieves various motions by arranging several actuators using accurate adjustment of large displacements in an intended direction.

## Supplementary Materials

The following are available online at https://www.mdpi.com/2072-666X/12/1/46/s1, Video S1: repeated operation of a standalone thermopneumatic actuator system.

## References

[1] Wehner M., Truby R.L., Fitzgerald D.J., Mosadegh B., Whitesides G.M., Lewis J.A., Wood R.J. (2016). An integrated design and fabrication strategy for entirely soft, autonomous robots. Nature.

[2] Mirvakili S.M., Hunter I.W. (2018). Artificial Muscles: Mechanisms, Applications, and Challenges. Adv. Mater..

[3] Rus D., Tolley M.T. (2015). Design, fabrication and control of soft robots. Nature.

[4] Yu Y.S., Zhao Y.P. (2009). Deformation of PDMS membrane and microcantilever by a water droplet: Comparison between Mooney-Rivlin and linear elastic constitutive models. J. Colloid Interface Sci..

[5] Bernacca G.M., O’Connor B., Williams D.F., Wheatley D.J. (2002). Hydrodynamic function of polyurethane prosthetic heart valves: Influences of Young’s modulus and leaflet thickness. Biomaterials.

[6] Boubakri A., Guermazi N., Elleuch K., Ayedi H.F. (2010). Study of UV-aging of thermoplastic polyurethane material. Mater. Sci. Eng. A.

[7] Case J.C., White E.L., Kramer R.K. (2015). Soft Material Characterization for Robotic Applications. Soft Robot..

[8] Calderón A.A., Ugalde J.C., Chang L., Cristóbal Zagal J., Pérez-Arancibia N.O. (2019). An earthworm-inspired soft robot with perceptive artificial skin. Bioinspir. Biomim..

[9] Mosadegh B., Polygerinos P., Keplinger C., Wennstedt S., Shepherd R.F., Gupta U., Shim J., Bertoldi K., Walsh C.J., Whitesides G.M. (2014). Pneumatic networks for soft robotics that actuate rapidly. Adv. Funct. Mater..

[10] Kawun P., Leahy S., Lai Y. (2016). A thin PDMS nozzle/diffuser micropump for biomedical applications. Sens. Actuators A Phys..

[11] Huh D., Matthews B.D., Mammoto A., Montoya-Zavala M., Yuan Hsin H., Ingber D.E. (2010). Reconstituting organ-level lung functions on a chip. Science.

[12] Kim H.J., Huh D., Hamilton G., Ingber D.E. (2012). Human gut-on-a-chip inhabited by microbial flora that experiences intestinal peristalsis-like motions and flow. Lab Chip.

[13] Wei H., Chueh B.H., Wu H., Hall E.W., Li C.W., Schirhagl R., Lin J.M., Zare R.N. (2011). Particle sorting using a porous membrane in a microfluidic device. Lab Chip.

[14] Hughes J., Culha U., Giardina F., Guenther F., Rosendo A., Iida F. (2016). Soft manipulators and grippers: A review. Front. Robot. AI.

[15] Nassar J.M., Khan S.M., Velling S.J., Diaz-Gaxiola A., Shaikh S.F., Geraldi N.R., Torres Sevilla G.A., Duarte C.M., Hussain M.M. (2018). Compliant lightweight non-invasive standalone “Marine Skin” tagging system. NPJ Flex. Electron..

[16] Stevens C., Powell D.E., Mäkelä P., Karman C. (2001). Fate and effects of polydimethylsiloxane (PDMS) in marine environments. Mar. Pollut. Bull..

[17] Slesarenko V., Engelkemier S., Galich P.I., Vladimirsky D., Klein G., Rudykh S. (2018). Strategies to Control Performance of 3D-Printed, Cable-Driven Soft Polymer Actuators: From Simple Architectures to Gripper Prototype. Polymers.

[18] Shintake J., Rosset S., Schubert B., Floreano D., Shea H. (2016). Versatile Soft Grippers with Intrinsic Electroadhesion Based on Multifunctional Polymer Actuators. Adv. Mater..

[19] An S., Kang D.J., Yarin A.L. (2018). A blister-like soft nano-textured thermo-pneumatic actuator as an artificial muscle. Nanoscale.

[20] Schift H. (2015). Nanoimprint lithography: 2D or not 2D? A review. Appl. Phys. A.

[21] Xia Y., Whitesides G.M. (1998). Soft lithography. Annu. Rev. Mater. Sci..

[22] Wu H., Odom T.W., Chiu D.T., Whitesides G.M. (2003). Fabrication of complex three-dimensional microchannel systems in PDMS. J. Am. Chem. Soc..

[23] Jung W., Kang Y., Han S., Hwang Y. (2019). Biocompatible micro, soft bellow actuator rapidly manufactured using 3D-printed soluble mold. J. Micromech. Microeng..

[24] Duggan T., Horowitz L., Ulug A., Baker E., Petersen K. Inchworm-inspired locomotion in untethered soft robots. Proceedings of the 2019 2nd IEEE International Conference on Soft Robotics (RoboSoft).

[25] Camino G., Lomakin S.M., Lazzari M. (2001). Polydimethylsiloxane thermal degradation Part 1. Kinetic aspects. Polymer.

[26] Kang K., Oh S., Yi H., Han S., Hwang Y. (2018). Fabrication of truly 3D microfluidic channel using 3D-printed soluble mold. Biomicrofluidics.

[27] Michalski D., Strąk K., Piasecka M. (2017). Comparison of two surface temperature measurement using thermocouples and infrared camera. EPJ Web Conf..

[28] Sun J.Y., Xia S., Moon M.W., Oh K.H., Kim K.S. (2012). Folding wrinkles of a thin stiff layer on a soft substrate. Proc. R. Soc. A Math. Phys. Eng. Sci..

[29] Kim T.K., Kim J.K., Jeong O.C. (2011). Measurement of nonlinear mechanical properties of PDMS elastomer. Microelectron. Eng..

[30] Liu M., Sun J., Chen Q. (2009). Influences of heating temperature on mechanical properties of polydimethylsiloxane. Sens. Actuators A Phys..

[31] Dong F., Sun X., Feng S. (2016). Thermal degradation kinetics of functional polysiloxanes containing chloromethyl groups. Thermochim. Acta.

[32] Xu S., Zhang H., Yu F., Zhao X., Wang Y. (2018). Enhanced ethanol recovery of PDMS mixed matrix membranes with hydrophobically modified ZIF-90. Sep. Purif. Technol..

[33] Sawano S., Naka K., Werber A., Zappe H., Konishi S. Sealing method of pdms as elastic material for mems. Proceedings of the 2008 IEEE 21st International Conference on Micro Electro Mechanical Systems.

[34] AbuZaiter A., Nafea M., Mohamed Ali M.S. (2016). Development of a shape-memory-alloy micromanipulator based on integrated bimorph microactuators. Mechatronics.

